# Penile Carcinoma Secondary to Balanitis Xerotica Obliterans and Its Compounding Resultant Pathologies: A Case Report

**DOI:** 10.7759/cureus.59555

**Published:** 2024-05-02

**Authors:** Ali Z Ansari, Sahar Hafeez, Joshua J Gallagher, Srihita Patibandla, Ali Saeed, Kurt Kratz

**Affiliations:** 1 Pathology, William Carey University College of Osteopathic Medicine, Hattiesburg, USA; 2 Pathology, Merit Health Wesley, Hattiesburg, USA

**Keywords:** posterior urethral stricture, catherization, risk factors of erectile dysfunction, meatoplasty, role of steroids, penile squamous cell carcinoma, male genitourinary system, male circumcision, chronic urinary retention, balanitis xerotica obliterans

## Abstract

A 57-year-old African-American male presented with urinary retention secondary to a history of balanitis xerotica obliterans (BXO) concurrent with penile carcinoma. BXO, characterized by chronic, sclerosing inflammation of the male external genitalia, presents significant clinical challenges due to its progressive nature and potential for complications. The patient experienced recurrent episodes of urinary retention, leading to multiple hospital visits and disease progression, prompting a comprehensive evaluation and intervention. The patient's medical history revealed a complex array of comorbidities, including penile carcinoma secondary to BXO, urethral strictures, and meatal stenosis. Clinical assessment, including bedside bladder ultrasound and laboratory investigations, confirmed urinary retention secondary to urethral stricture, necessitating urological consultation. Management strategies involved Foley catheter placement, urethral dilation, and pharmacological interventions for pain management. Subsequent follow-up and imaging evaluations identified an increased risk of carcinoma development, highlighting the importance of surveillance and early intervention in patients with BXO. This case report highlights the intricate clinical manifestations and therapeutic considerations encountered in managing BXO and its associated pathologies.

## Introduction

Balanitis xerotica obliterans (BXO) is a chronic and progressive inflammatory condition affecting the male external genitalia. Initially termed lichen sclerosis of the glans penis and prepuce by Hallopeau in 1887, BXO presents notable clinical complexities [1]. It predominantly affects the prepuce, frenulum, glans, external urethral meatus, and peripheral urethra, with potential progression to involve the entire urethra, penile skin, and scrotum if left untreated. Common symptoms include itching, pain, and inflammation, leading to the development of white, thickened, and scarred lesions over time [2].

The etiology of BXO remains unclear, complicating its management and treatment. However, its progression can lead to urethral stenosis and urinary retention in advanced stages. As such, early recognition and intervention are paramount to mitigate potential complications. The diagnosis of BXO typically involves a clinical examination, which may reveal characteristic white, parchment-like plaques. This diagnosis may be supported by histopathological findings on biopsy, demonstrating dermal fibrosis and lymphocytic infiltration. This treatment usually consists of applying topical corticosteroid creams to reduce inflammation and itching [3]. Severe cases with substantial urethral scarring might require surgical procedures like circumcision or meatotomy to restore proper urinary function. When advanced cases remain unnoticed or untreated, they can lead to compounded pathologies.

Urinary retention, the inability to expel urine adequately, poses a complex urological challenge, predominantly affecting males. It stems from various causes such as benign prostatic hyperplasia (BPH), neurological issues, iatrogenic factors, and structural abnormalities [4]. While BPH accounts for about 53% of cases, a thorough understanding of all potential causes is essential [5]. Diagnosis involves detailed medical history, physical examination, including neurologic assessment, and measuring postvoid residual (PVR) urine volume. As characterized by the American Urological Association, chronic urinary retention is defined as a PVR volume exceeding 300 mL on two occasions over six months. Management begins with prompt urethral catheterization for complete bladder decompression. Suprapubic catheters offer advantages like patient comfort and reducing bacteriuria [6]. Further management depends on identifying the underlying cause, potentially including alpha-blockers and close monitoring by neurology and urology specialists for neurologic causes.

A urinary stricture, or urethral stricture, is a pathological narrowing of the urethra, the anatomical structure responsible for transporting urine from the bladder out of the body. Etiologically, urinary strictures can arise from a spectrum of causes including inflammatory processes, traumatic injuries, iatrogenic interventions, and congenital abnormalities. Inflammatory conditions such as urethritis, often triggered by infectious agents or autoimmune processes, can lead to the formation of scar tissue within the urethra and result in further chronic narrowing [7]. Traumatic events such as pelvic fractures or instrumentation-related injuries, such as frequent catheterizations, meatal dilations, or urethral surgeries, may also cause tissue damage and subsequent scarring, leading to strictures. Moreover, conditions such as BXO can predispose individuals to the development of strictures later in life, highlighting the importance of a thorough patient history. This case report aims to thoroughly explore the clinical manifestations and treatment considerations in managing BXO.

## Case presentation

A 57-year-old African-American male presented to the emergency department (ED) with a chief complaint of urinary retention, a recurring issue he had experienced over the past two years. He attributed the current episode to difficulty urinating after a day spent working in his yard, prompting his decision to seek evaluation in the ED. On initial examination, the patient appeared alert and comfortable, displaying no signs of acute distress. His skin was warm and dry, and he was saturating at 100% on room air. The genital examination revealed the presence of white, sclerotic plaques on the foreskin and glans penis.

The patient's medical history was notable for a complex array of urological conditions, including recurrent urethral strictures, meatal stenosis, and BXO, which occurred concurrently with penile carcinoma. Over the past decade, he had undergone multiple surgical interventions, including circumcision, cystoscopy, meatoplasty, meatotomy, urethral dilation, and urethroplasty. Notably, a previous distal urethroplasty resulted in hypospadias. His symptoms of urinary retention were temporarily alleviated until their recurrence two years ago. Additionally, the patient received treatment for genital warts, including topical application of podophyllin or imiquimod cream, as well as cryotherapy. These interventions were effective in eliminating the warts, with no documented recurrence observed during subsequent follow-up appointments.

**Figure 1:**
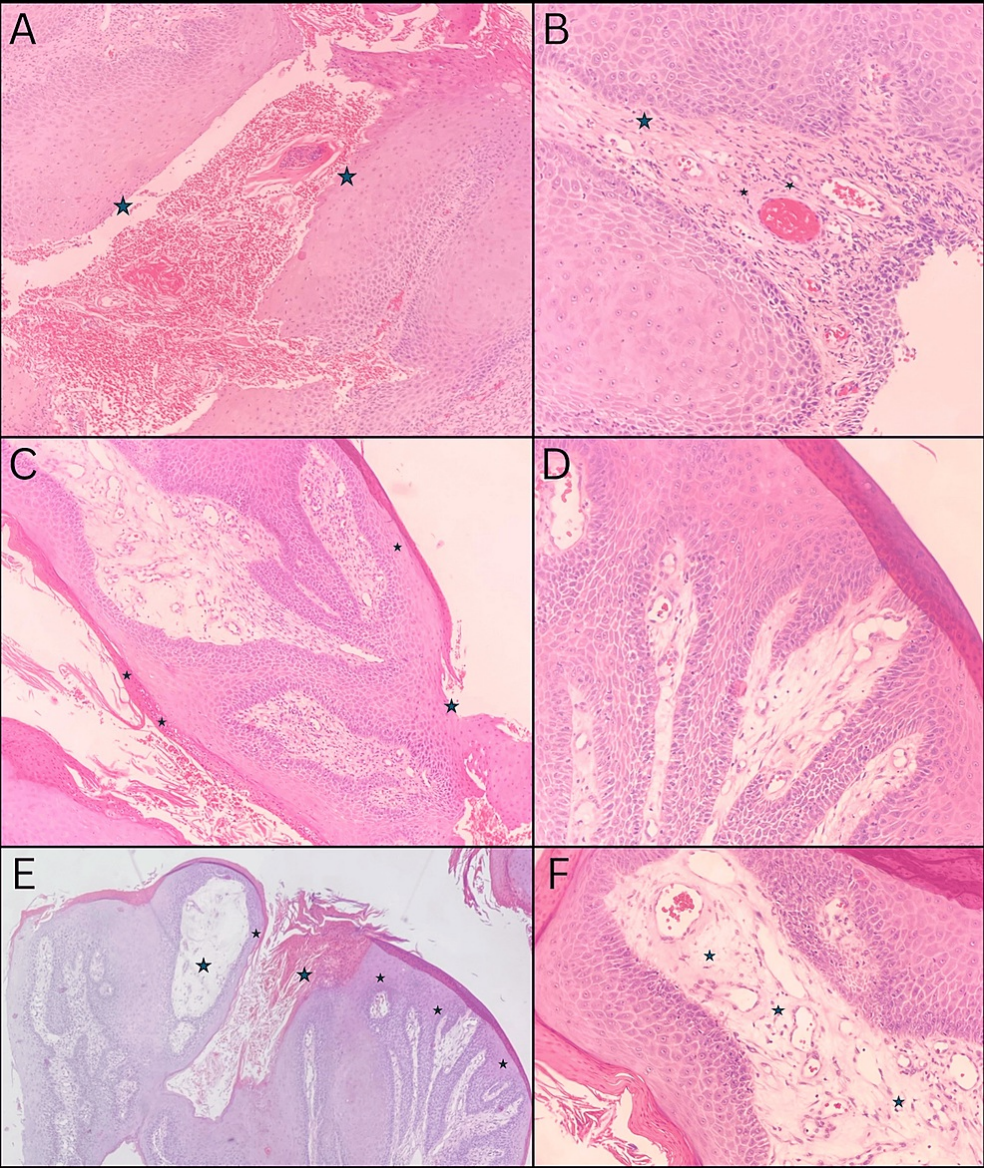
Histological photomicrograph of penile squamous cell carcinoma stained with hematoxylin and eosin. A: A 40x magnification of late-stage epithelial ulceration is evident by tissue separation at the basal layer. B: A 200x magnification of distinctive perivascular hyalinization (small stars) and lymphocytic depletion (big star) at the epithelium-lamina propria interface, characteristic of cancer-associated lichen sclerosus. C: A 100x magnification of linear sclerosis of the basement membrane, accompanied by basal cell vacuolization. Notably, the epithelium displays significant thinning and ulceration. D: A 200x magnification of hyperplastic squamous epithelium, exhibiting well-differentiated cancer cells with an intact basement membrane. Noteworthy are the aberrant nucleus-cytoplasmic ratio and frequent occurrence of mitotic figures. E: A 10x magnification of marked edema and hyalinization of the lamina propria (big stars), accompanied by epithelial thinning and denudation (small stars). F: A 200x magnification of thickening of the lamina propria, is characterized by hypervascularization and loss of structural integrity.

The patient reported a longstanding history of BXO spanning 12 years and noted the development of a mass on the head of his penis two years prior to his current presentation. Approximately one year before presenting to the ED with similar complaints of urinary retention, a biopsy of the penile mass was performed due to concerns of carcinoma development. The histological examination confirmed the presence of squamous cell carcinoma (Figure 1), prompting surgical resection.

He notified the ED staff that following his surgery, he had experienced occasional urinary retention requiring Foley catheter insertion. However, earlier that day, he had managed to urinate normally. Concerns arose about dehydration potentially contributing to his condition, as the patient denied any other relevant factors. This prompted a bedside bladder ultrasound scan, revealing a bladder volume of 159.6 mL, notably lower than the typical healthy range of 300 to 400 mL [8]. Given the calculated bladder volume, the patient was given oral liquids to increase urinary output. After rehydration of the patient, another scan was taken to re-evaluate. The patient was then encouraged to attempt voiding his bladder after consuming water.

**Figure 2:**
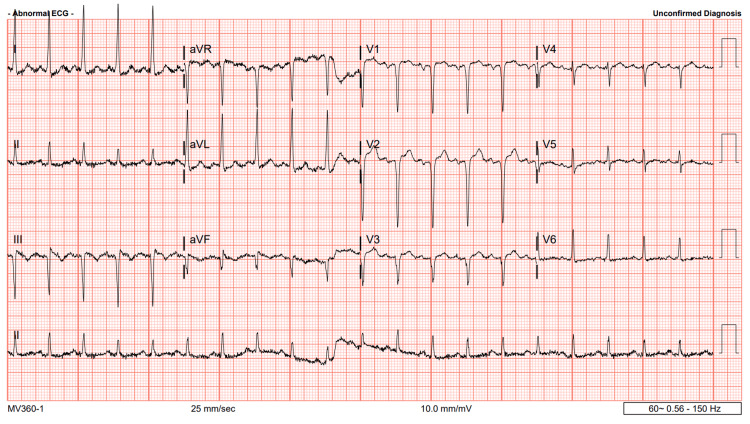
EKG findings reveal sinus tachycardia and moderate voltage criteria suggestive of left ventricular hypertrophy, alongside noted inferior and anteroseptal infarcts of uncertain duration. EKG: electrocardiogram

Following unsuccessful attempts to induce urination, it was decided to proceed with Foley catheter placement. The patient disclosed a history of previous success with catheterization. Prior to the procedure, the patient received dihydrocodeine for comfort. Despite multiple attempts, including the use of the smallest 8-French gauge Foley catheter, catheterization was not successful. Urology consultation was sought, and the patient was advised to be started on alpha-blockers due to suspected prostatic enlargement. During reassessment, the patient's blood pressure was found to be elevated at 226/111 mmHg, attributed to pain and discomfort from the Foley attempts. The patient denied any prior hypertensive episodes or awareness of elevated blood pressure. In response, morphine and Zofran were administered to alleviate discomfort, while an electrocardiogram (EKG) and chest X-ray were ordered. The EKG indicated sinus tachycardia with moderate voltage criteria for left ventricular hypertrophy, along with noted inferior and anteroseptal infarcts of indeterminate age (Figure 2). The chest X-ray findings were unremarkable. Later, the patient experienced bladder spasms and a heightened urgency to urinate.

For further evaluation, a second bladder scan was ordered. Subsequent examination indicated an increased bladder volume of 490 mL, compared to the reading obtained four and a half hours earlier hours earlier. This prompted additional consultation with the urology team. Upon evaluation by the urologist, a successful urethral dilation was performed as per their recommendation, followed by the insertion of a 14-French silicone catheter. Further investigation revealed no acute abnormalities in laboratory results or imaging studies. The patient reported feeling relieved after the catheter placement. Long-term follow-up arrangements were made, with the patient scheduled for regular urology appointments to determine the necessity for any additional interventions. Upon discharge, the patient received comprehensive instructions for follow-up care, along with a prescription for cephalosporin. Additionally, the patient was counseled to promptly return to the ED if symptoms showed any signs of worsening.

## Discussion

While precise statistics on the prevalence of BXO can vary depending on the population studied and diagnostic criteria used, it is generally considered to be a relatively rare condition. BXO is more commonly diagnosed in males, particularly in those who are uncircumcised. However, the exact prevalence is not well-established. The shortcomings in statistical evidence make it difficult to understand the progression of this disorder, but it's estimated to affect around 0.1% to 0.3% of the male population [9]. A study found that out of a total of 153,432 male patients, 108 individuals (0.070%) received a diagnosis of BXO. The age distribution displayed consistency across a broad spectrum, ranging from 2 to 90 years, with the exception of the third decade, where the incidence nearly doubled. Notably, it was observed that Black and Hispanic patients demonstrated an approximately twofold higher incidence rate in comparison to White patients, with respective rates of 10.59, 10.67, and 5.07 cases per 10,000 patients [10].

Due to its rarity, it is important to recognize its potential impact on affected individuals. The condition can lead to significant discomfort, pain, and functional impairment, particularly due to its effects on the genitourinary system. There is also a small risk of malignant transformation, particularly in cases where BXO involves the genitalia as seen in the case presented. Early diagnosis and appropriate management are essential in managing BXO and minimizing its impact on affected individuals. This treatment typically involves the use of topical corticosteroids to reduce inflammation and symptoms. In cases where BXO leads to complications such as urinary strictures or sexual dysfunction, surgical intervention may be necessary. However, while surgical intervention can be beneficial for disease management, it is important to acknowledge the significant challenges posed by patients.

One primary concern involves the discomfort and postoperative pain associated with surgical interventions for BXO, including urethral dilatation, meatoplasty, or reconstructive surgeries, which can lead to a lengthy recovery period marked by swelling, discomfort, and limitations on daily activities and sexual lifestyles. Patients may face emotional challenges due to feelings of humiliation or disfigurement in intimate areas, highlighting the importance of sensitivity and respect in healthcare discussions. It's crucial for healthcare providers to thoroughly discuss surgical risks, benefits, and alternatives, such as topical corticosteroids or immunomodulatory therapies, based on individual patient preferences and the severity of the condition.

In addition to surgical considerations, it is important to address the potential acceleration and exacerbation of concurrent disease processes, particularly carcinoma development. The patient presented a complex medical history, notably featuring an atypical well-differentiated squamous proliferation originating from a small mass on the head of the penis. Additionally, the patient has a medical history of genital warts, further increasing the risk of carcinogenesis. The identified carcinomas displayed distinctive features, notably lacking verrucous characteristics attributed to the involvement of the specimen base.

It is pertinent to acknowledge the heightened risk of carcinoma development associated with chronic inflammation [11]. The correlation between penile cancer and BXO was originally underestimated; however, recent literature analysis indicates approximately 28% to 50% of penile cancer patients have a history of BXO [12]. It is also estimated that the risk of squamous cell carcinoma development due to BXO is 2% to 15% [13]. The development of phimosis is recognized as a risk factor for the proliferation of penile cancer, suggesting that circumcision may act as a mitigating factor in the disease process [14]. This indication becomes an important discussion point when noting that tumor-specific mortality is 30%, and circumcision can reduce the risk to a hazard level of 0.33 [15]. Recognizing the effectiveness of steroid creams in limiting disease progression and understanding that early circumcision can offer a cure, highlights how timely interventions can offer life-changing impact for those with BXO [16].

## Conclusions

This case report highlights the need for raising awareness about BXO among healthcare professionals and affected individuals alike. Early diagnosis and appropriate treatment are vital to prevent the potentially severe complications associated with BXO, such as urinary strictures, sexual dysfunction, and an increased risk of penile cancer. Bringing attention to cases of BXO allows healthcare professionals to facilitate prompt intervention, ultimately improving patient outcomes and well-being. Education initiatives aimed at dispelling misconceptions and providing support for emotional distress are equally essential for both patients and healthcare providers. Moreover, increased awareness of BXO can drive research efforts toward better understanding its underlying causes and optimal treatment strategies, fostering advancements in diagnosis and care. Through collaborative efforts and enhanced understanding, we can work toward improving the management of BXO and enhancing the quality of life for affected individuals.
